# p53 alterations in recurrent squamous cell cancer of the head and neck refractory to radiotherapy

**DOI:** 10.1054/bjoc.1999.0932

**Published:** 2000-01-18

**Authors:** I Ganly, D S Soutar, R Brown, S B Kaye

**Affiliations:** Dept of Head and Neck Plastic and Reconstructive Surgery, Canniesburn Hospital, Switchback Road, Bearsden, UK; CRC Dept of Medical Oncology, Garscube Estate, Switchback Road, Bearsden, UK

**Keywords:** p53, HPV, mdm2, head and neck cancer

## Abstract

The aim of the study was to determine the incidence of p53 alterations by mutation, deletion or inactivation by mdm2 or human papillomavirus (HPV) infection in recurrent squamous cell cancer of the head and neck (SCCHN) refractory to radiotherapy. Twenty-two tumours were studied. The p53 status of each tumour was analysed by sequencing of exons 4–9 and by immunohistochemistry. Mdm2 expression was assessed by immunohistochemistry and HPV infection was assessed by polymerase chain reaction of tumour DNA for HPV 16, 18 and 33. Fifteen (68%) of the 22 tumours studied had p53 mutations, while seven had wild-type p53 sequence. p53 immunohistochemistry correlated with the type of mutation. HPV DNA was detected in 8 (36%) tumours and all were of serotype HPV 16. Of these, five were in tumours with mutant p53 and three were in tumours with wild-type p53. Mdm2 overexpression was detected in 11 (50%) tumours. Of these, seven were in tumours with mutant p53 and four were in tumours with wild-type p53. Overall, 21 of the 22 tumours had p53 alterations either by mutation, deletion or inactivation by mdm2 or HPV. In this study, the overall incidence of p53 inactivation in recurrent head and neck cancer was very high at 95%. The main mechanism of inactivation was gene mutation or deletion which occurred in 15 of the 22 tumours studied. In addition, six of the seven tumours with wild-type p53 sequence had either HPV 16 DNA, overexpression of mdm2 or both which suggested that these tumours had p53 inactivation by these mechanisms. This high incidence of p53 dysfunction is one factor which could account for the poor response of these tumours to radiotherapy and chemotherapy. Therefore, new therapies for recurrent SCCHN which either act in a p53 independent pathway, or which restore p53 function may be beneficial in this disease. © 2000 Cancer Research Campaign


*British Journal of Cancer* (2000) **82**(2), 392–398
© 2000 Cancer Research Campaign
Article no. bjoc.1999.0932

### p53 alterations in recurrent squamous cell cancer of the head and neck refractory to radiotherapy

**I Ganly[1,2], DS Soutar[1], R Brown[2] and SB Kaye[2]**

[1]Dept of Head and Neck Plastic and Reconstructive Surgery, Canniesburn Hospital, Switchback Road, Bearsden, Glasgow, UK; [2]CRC Dept of Medical Oncology, Garscube Estate, Switchback Road, Bearsden, Glasgow, UK

**Summary** The aim of the study was to determine the incidence of p53 alterations by mutation, deletion or inactivation by mdm2 or human papillomavirus (HPV) infection in recurrent squamous cell cancer of the head and neck (SCCHN) refractory to radiotherapy. Twenty-two tumours were studied. The p53 status of each tumour was analysed by sequencing of exons 4–9 and by immunohistochemistry. Mdm2 expression was assessed by immunohistochemistry and HPV infection was assessed by polymerase chain reaction of tumour DNA for HPV 16, 18 and 33. Fifteen (68%) of the 22 tumours studied had p53 mutations, while seven had wild-type p53 sequence. p53 immunohistochemistry correlated with the type of mutation. HPV DNA was detected in 8 (36%) tumours and all were of serotype HPV 16. Of these, five were in tumours with mutant p53 and three were in tumours with wild-type p53. Mdm2 overexpression was detected in 11 (50%) tumours. Of these, seven were in tumours with mutant p53 and four were in tumours with wild-type p53. Overall, 21 of the 22 tumours had p53 alterations either by mutation, deletion or inactivation by mdm2 or HPV. In this study, the overall incidence of p53 inactivation in recurrent head and neck cancer was very high at 95%. The main mechanism of inactivation was gene mutation or deletion which occurred in 15 of the 22 tumours studied. In addition, six of the seven tumours with wild-type p53 sequence had either HPV 16 DNA, overexpression of mdm2 or both which suggested that these tumours had p53 inactivation by these mechanisms. This high incidence of p53 dysfunction is one factor which could account for the poor response of these tumours to radiotherapy and chemotherapy. Therefore, new therapies for recurrent SCCHN which either act in a p53 independent pathway, or which restore p53 function may be beneficial in this disease. © 2000 Cancer Research Campaign

**Keywords:** p53; HPV; mdm2; head and neck cancer

Local recurrence is the most common cause of failure after head and neck cancer surgery and the most important factor which predisposes to recurrence is incomplete surgical resection margins (Snow et al, 1989; Jones et al, 1992). Recurrent squamous cell cancer of the head and neck (SCCHN) causes significant morbidity including effects on speech and swallowing. It is a disease with a very poor prognosis as currently available therapies including re-irradiation (Nickers, 1997) and chemotherapy (Clavel, 1994) all have poor response rates which are short lasting (Forestiere, 1994). The reason for this is unclear but is likely to be multifactorial in nature. One factor which may be important in resistance to therapy is loss of function of the tumour suppressor gene p53. It has been shown that inactivation of the p53 tumour suppressor gene is one of the major predictors of failure to respond to radiotherapy and chemotherapy in many tumour types (McIlwrath et al, 1994; Buttitta et al, 1997; Cutilli et al, 1998). This is because p53 plays a major role in the induction of apoptosis in response to genotoxic agents such as radiotherapy and chemotherapy (Lowe et al, 1993; Huang et al, 1996). Therefore one possible explanation for the poor response rates in recurrent SCCHN to radiotherapy and chemotherapy could be due to a high incidence of p53 inactivation in this disease. No previous studies have been reported on the incidence or mechanism of p53 inactivation in recurrent head and neck cancer. The objectives of this study were therefore to determine the incidence of p53 mutations in this disease and also gain insight into other mechanisms of p53 inactivation such as binding by the cellular protein mdm2 (Oliner et al, 1992; Haupt et al, 1997) and viral protein HPV E6 (Scheffner et al, 1990; Lechner et al, 1992). To determine the incidence of p53 inactivation in recurrent HNSCC, 22 recurrent tumours from patients previously treated with radiotherapy ± surgery were analysed. The p53 status of each tumour was analysed by sequencing and by immunohistochemistry. Tumours were further analysed for mdm2 overexpression by immunohistochemistry and for human papillomavirus (HPV) infection by polymerase chain reaction (PCR) of tumour DNA. Overall we have shown that there is a greater incidence of p53 mutations in recurrent disease compared to primary disease. However, the incidence of HPV infection and mdm2 overexpression was similar to reported studies in primary disease. Nevertheless, we show that the overall incidence of p53 alterations is very high at 95%. This may be one factor which accounts for the poor response of this disease to radiotherapy and chemotherapy and implies new therapies which either restore p53 function or which act in a p53 indpendent manner may prove to be beneficial in this disease.

*Received 6 May 1999*
*Revised 28 July 1999*
*Accepted 3 August 1999*

*Correspondence to:* I Ganly, CRC Dept of Medical Oncology, CRC Beatson Laboratories, Garscube Estate, Switchback Road, Glasgow G61 1BD, UK

### METHODS

### Tumour collection

Patients with recurrent SCCHN gave consent for biopsies to be taken from the recurrent tumours. Core biopsies were taken using a 14 gauge tru-cut needle under local anaesthetic. One sample was snap-frozen in liquid nitrogen for DNA extraction. One sample was fixed in phosphate-buffered formalin, embedded in paraffin from which 5-μm sections were cut for immunohistochemical analysis.

### Immunohistochemistry

### *Immunohistochemistry for p53*

Immunohistochemistry was carried out on formalin-fixed paraffin-embedded tumours cut into 5-μm sections. Slides were deparaffinized in xylene, hydrated through ethanols 100%, 90%, 70% and then water, then washed in PBS (phosphate-buffered saline). Antigen retrieval was carried out by microwaving in citrate buffer pH 6.0 at 500 W for 25 min and then allowed to cool over 20 min. The slides were washed in PBS for 5 min and then endogenous peroxide activity blocked with 3% v/v hydrogen peroxide in methanol for 10 min. After washing in PBS for 5 min, the slides were blocked with Universal blocking solution (Biogenex) for 10 min, and then primary antibody (DO-1 Oncogene Science) at a dilution of 1/1000 in Dako antibody diluent solution, added for 1 h at room temperature. Antigen detection was done using a biotinylated second antibody followed by streptavidin as supplied in the Biogenex link/label kit. The chromogen used for detection was diaminobenzidine (Vector) for 3–10 min. The sections were counterstained with haematoxylin, dehydrated in graded alcohols followed by xylene, and then mounted in DPX mounting medium (BDH Chemicals). The immunohistochemistry pattern was scored using a histoscore based on intensity of staining and percentage of positive cells staining and given a score out of a maximum of 6 (Table 1).

**Table 1**

| Intensity of staining | Score | Cells staining positive (%) | Score |
|---|---|---|---|
| None | 0 | 0 | 0 |
| Mild | 1 | 5–20 | 1 |
| Moderate | 2 | 20–80 | 2 |
| Severe | 3 | > 80 | 3 |

### *Immunohistochemistry for mdm2*

The protocol used was as described for p53 immunohistochemistry. The primary antibody used was a mouse monoclonal antibody SMP14 (Dako) at a dilution of 1/50 in PBS. Mdm2 staining was then scored by the % of cells staining positive and by the intensity of staining as described above.

### DNA extraction

Genomic DNA was isolated directly from frozen tumours by lysis buffer containing 10 mM Tris–HCl, 10 mM EDTA, 10 mM sodium chloride, 4% N-lauryl sarcosine and 2.75 mg $ml^{-1}$ proteinase K, followed by overnight digestion at 55°C and EtOH precipitation. The resulting pellet was washed with 70% EtOH, dried, and resuspended in 100 μl TE and stored at 4°C.

### p53 sequencing

Exons 4 to 9 were sequenced using DNA extracted from pre-treatment tumour biopsies. This was done by Oncormed Corporation, Gaithersburg, Maryland.

### HPV analysis

The presence of HPV in tumour DNA was assessed by PCR. The primers for the amplification of HPV 16 and HPV 18 have been described previously (Yeudall et al, 1991) and amplify fragments of the E6/E7 (HPV 16) or E6 regions (HPV 18), which are 165 and 99 bp respectively. HPV 33 primers were those as described by Haraf et al (1996).

**Table 2** Primary tumour, site of recurrence and previous treatment

| Pt no. | Sex | Age | Primary | Site of recurrence | Prior therapy | | |
|---|---|---|---|---|---|---|---|
| | | | | | Chemotherapy | Radiotherapy | Surgery |
| 1 | M | 61 | Piriform fossa | Left cervical | Yes | Yes | No |
| 2 | M | 74 | Hypopharynx | Bilateral cervical | Yes | Yes | No |
| 3 | M | 32 | Tongue | Right submandibular | No | Yes | Yes |
| 4 | F | 80 | Palate | Right clavicular area | No | Yes | Yes |
| 5 | F | 68 | Floor of mouth | Right neck-post triangle | No | Yes | Yes |
| 6 | F | 38 | Left auditory canal | Left cervical | Yes | Yes | No |
| 7 | M | 53 | Left temple | Left preauricular area | No | Yes | Yes |
| 8 | F | 72 | Right retromolar trigone | Right cervical | No | Yes | No |
| 9 | M | 56 | Floor of mouth | Left submandibular | No | Yes | Yes |
| 10 | M | 88 | Left palatoglossal fold | Bilateral cervical | No | Yes | Yes |
| 11 | M | 76 | Supraglottic larynx | Left cervical | No | Yes | No |
| 12 | M | 82 | Retromolar trigone | Left cervical | No | Yes | No |
| 13 | M | 62 | Tongue | Left cervical | No | Yes | Yes |
| 14 | M | 75 | Supraglottic larynx | Left submandibular | No | Yes | Yes |
| 15 | M | 60 | Larynx | Right cervical | No | Yes | Yes |
| 16 | F | 64 | Piriform fossa | Right cervical | Yes | Yes | No |
| 17 | M | 59 | Oropharynx | Left clavicular | No | Yes | Yes |
| 18 | F | 58 | Supraglottic larynx | Left cervical | No | Yes | Yes |
| 19 | F | 59 | Oropharynx | Left cervical | No | Yes | Yes |
| 20 | M | 51 | Tongue | Right cervical | No | Yes | Yes |
| 21 | F | 75 | Post cricoid | Left cervical | No | Yes | No |
| 22 | M | 65 | Supraglottic larynx | Left supraclavicular | No | Yes | Yes |

© 2000 Cancer Research Campaign 

The HPV16 primers were: 5′-TTAATTAGGTGTATTAACTG-3′ and 5′-TGCATGATTACAGCRGGGTT-3′. The HPV18 primers were: 5′-ATCTGTGTGCACGGAACTAAC-3′ and 5′-AATGCA-AATTCAAATACCTC-3′. The HPV33 primers were: 5′-GTGC-CAAGCATTGGAGACAA-3′ and 5′-GATAAGAACCGCAAAC -ACAG-3′.

SiHa DNA (single copy HPV 16) and HeLa DNA (HPV 18) were used as positive controls. HPV amplification was carried out at 95°C for 3 min, then 30 cycles of 95°C for 30 s, 55°C for 30 s, 72°C for 30 s, followed by a final extension time of 10 min at 72°C. An aliquot was run on a 1% agarose gel (Nu-Sieve) containing 0.5 μg $ml^{-1}$ ethidium bromide in TBE buffer and then photographed under UV light to check for correct amplification.

**Table 3** p53 sequence of recurrent squamous cell cancers studied

| Pt no. | Exon | Codon | Base-pair change | Amino acid change | p53 Gene sequencing (Exons 4–9) |
|---|---|---|---|---|---|
| 1 | 5 | 376 | Splice site mutation | Truncated protein | Mutant-non-sense |
| 2 | 8 | 273 | CGT to TGT | Arginine to cysteine | Mutant-mis-sense |
| 3 | 8 | 307 | Deletion G | Stop signal | Mutant-non-sense |
| 4 | 6 | | 51 splice site mutn AG to AA | Truncated protein | Mutant-non-sense |
| 5 | 5 | 141 | TGC to TAC | Cysteine to tyrosine | Mutant-mis-sense |
| 6 | 9 | 336 | Frameshift mutation | Stop signal | Mutant-non-sense |
| 7 | 8 | 280 | AGA to ATA | Arginine to isoleucine | Mutant-mis-sense |
| 8 | 8 | 282 | CGG to TGG | Arginine to tryptophan | Mutant-mis-sense |
| 9 | wt | | | | Wild type |
| 10 | wt | | | | Wild type |
| 11 | wt | | | | Wild type |
| 12 | 8 | 266 | GGA to TGA | Glycine to stop signal-truncated protein | Mutant-non-sense |
| 13 | 6 | 205 | TAT to TGT | Tyrosine to cysteine | Mutant-mis-sense |
| 14 | wt | | | | Wild type |
| 15 | 7 | 233 | 11 bp insertion | Stop signal at codon 250 | Mutant-non-sense |
| 16 | 9 | 317 | CAG to TAG | Glutamine to stop signal-truncated protein | Mutant-non-sense |
| 17 | wt | | | | Wild type |
| 18 | 6 | 222 | Deletion G | Stop signal | Mutant-non-sense |
| 19 | wt | | | | Wild type |
| 20 | 5 | 163 | TAC to TGC | Tyrosine to cysteine | Mutant-mis-sense |
| 21 | wt | | | | Wild type |
| 22 | 5 | 167 | 10 bp deletion | Stop signal | Mutant-non-sense |

Of 22 tumours studied, 15 had mutant p53 and 7 had wild type p53. Of the mutant p53 sequences, 6 were missense mutations and 9 were nonsense mutations.

**Table 4** p53 and mdm2 immunohistochemistry histoscores and HPV PCR analysis in squamous cell cancers studied

| Patient no. | p53 gene seq. | p53 IHC | p53 histoscore | mdm2 IHC | mdm2 histoscore | HPV16 | HPV18/33 |
|---|---|---|---|---|---|---|---|
| 1 | Mutant-mis-sense | – | 0 | + | 2 | – | – |
| 2 | Mutant-non-sense | + | 4 | – | 0 | – | – |
| 3 | Mutant-non-sense | – | 0 | – | 0 | + | – |
| 4 | Mutant-mis-sense | – | 0 | + | 2 | – | – |
| 5 | Mutant-non-sense | + | 6 | – | 0 | – | – |
| 6 | Mutant-mis-sense | + | 6 | + | 4 | – | – |
| 7 | Mutant-mis-sense | + | 6 | – | 0 | – | – |
| 8 | Mutant-mis-sense | + | 5 | + | 2 | – | – |
| 9 | Wild type | + | 5 | + | 3 | – | – |
| 10 | Wild type | – | 0 | – | 0 | + | – |
| 11 | Wild type | – | 0 | + | 5 | – | – |
| 12 | Mutant-non-sense | – | 0 | + | 3 | + | – |
| 13 | Mutant-mis-sense | + | 4 | – | 0 | + | – |
| 14 | Wild type | + | 2 | – | 0 | – | – |
| 15 | Mutant-non-sense | – | 0 | – | 0 | + | – |
| 16 | Mutant-non-sense | – | 0 | – | 0 | + | – |
| 17 | Wild type | + | 2 | + | 5 | + | – |
| 18 | Mutant-non-sense | – | 0 | + | 4 | – | – |
| 19 | Wild type | – | 0 | – | 0 | + | – |
| 20 | Mutant-mis-sense | + | 4 | – | 0 | – | – |
| 21 | Wild type | + | 6 | + | 5 | – | – |
| 22 | Mutant-non-sense | – | 0 | + | 4 | – | – |

p53 immunohistochemistry histoscore correlated with the type of gene sequence; non-sense mutations had low histoscores and mis-sense mutations had high histoscores. Mdm2 protein expression was detected in 11 tumours and HPV16 detected in eight tumours. HPV18 and 33 DNA were not detected in any tumour.

 *© 2000 Cancer Research Campaign*

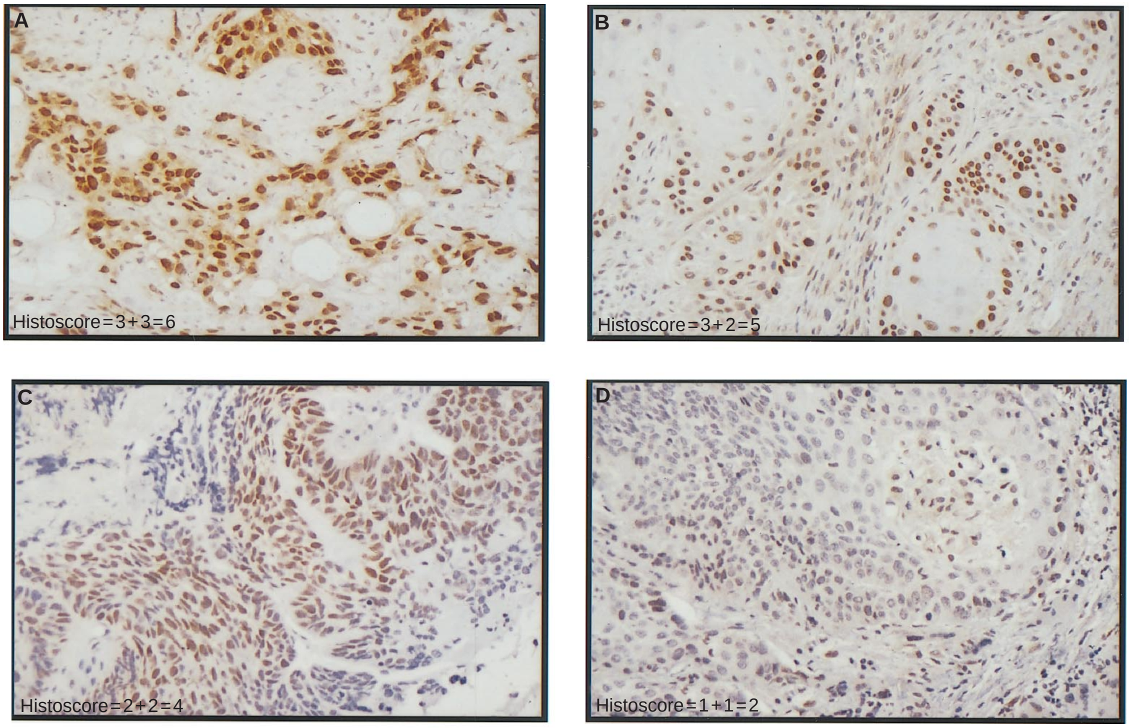

**Figure 1** Examples of p53 immunohistochemistry staining in SCCHN biopsies. Staining was graded using a histoscore grading system based on the intensity of staining and the percentage of cells staining positively as described in Methods

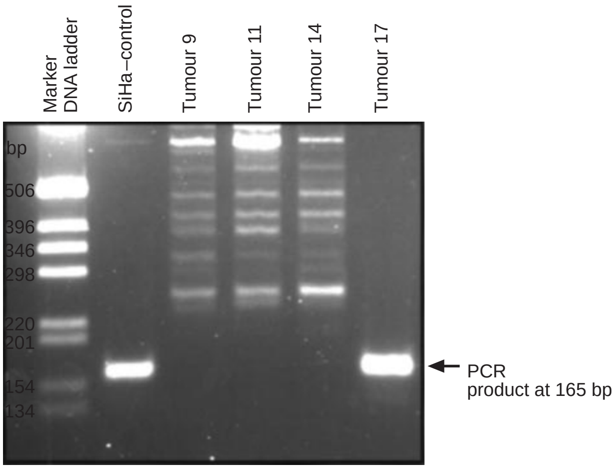

**Figure 2** HPV 16 E6/7 analysis of tumour DNA by PCR. DNA from SiHa cell line was used as a positive control for HPV16 E6/7. Tumour 17 is positive for HPV 16 with the correct PCR product at 165 base pairs

### RESULTS

### Primary tumour, site of recurrence and previous treatment

The primary tumour, site of recurrence and previous therapy for each patient studied are shown in Table 2. The mean age was 64 and the male to female ratio was 14:8. All patients had received radiotherapy. In addition, four patients had received chemotherapy and 14 patients had had surgery. The site of tumour recurrence was most commonly regional in the site of draining lymph nodes.

### Incidence of p53 mutation in recurrent SCCHN is high and type of mutation correlates with immunohistochemistry

The p53 sequence and immunohistochemistry for each tumour is shown in Tables 3 and 4. Of the 22 tumours studied, 15 had mutations in the *p53* gene of which six were mis-sense mutations and nine were non-sense mutations. Generally, the immunohistochemistry pattern correlated directly with the type of p53 mutation, i.e. mis-sense mutations gave high scores and non-sense mutations gave low scores. One patient (patient 6) had a non-sense mutation yet stained positively by immunohistochemistry. The site of the mutation in this patient's *p53* gene was in codon 9 and this would account for its detection by immunohistochemistry using the DO-1 antibody. Of the seven patients with wild-type p53 sequence, five were negative on immunohistochemistry (tumours 10, 11, 14, 17, 19) and two were positive indicating overexpression of wild-type p53 (tumours 9, 21). Examples of p53 immunohistochemistry with the relevant histoscores is shown in Figure 1.

### Incidence of HPV infection and mdm2 overexpression

Eight tumours were positive for HPV DNA, all of which were serotype HPV 16. Of these, five were in tumours with mutant p53 and three were in tumours with wild-type p53. A typical positive PCR reaction for HPV 16 is shown in Figure 2.

Mdm2 expression was detected in 11 of the 22 tumours (50%). Of these, five were weakly positive (histoscore = 2, 3) and six were strongly positive (histoscore = 4, 5, 6). Mdm2 expression was present in seven tumours with mutant p53 and in four tumours with wild-type p53.

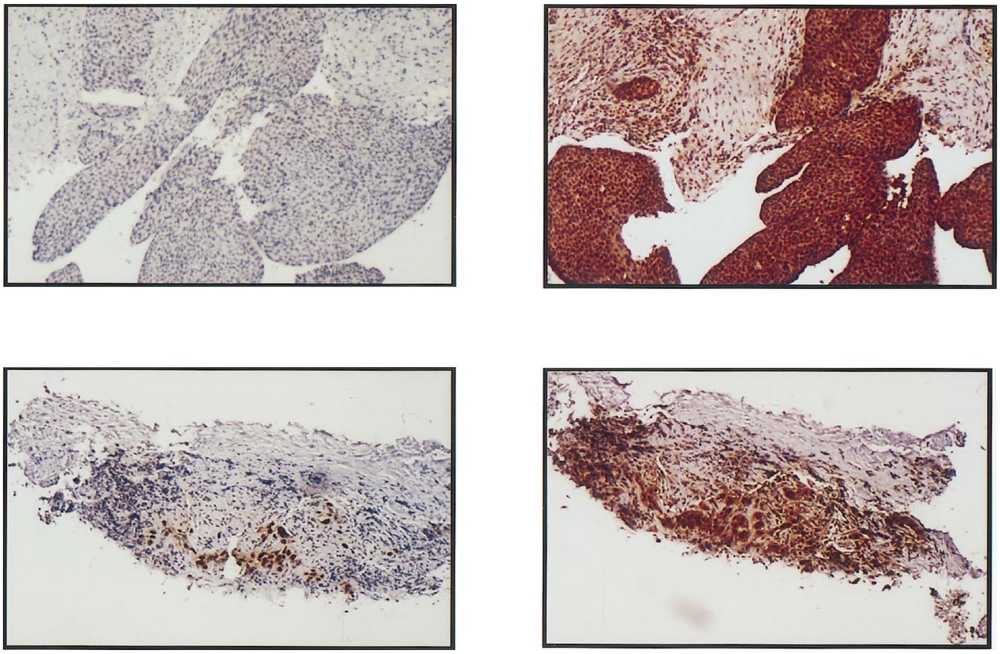

**Figure 3** Examples of p53 and mdm2 immunohistochemistry in tumours with wild-type p53 gene sequence. Tumour 17 has weak p53 staining but intense mdm2 staining suggesting p53 inactivation by mdm2 overexpression. Tumour 21 has overexpression of wild type p53 and also intense mdm2 staining suggesting p53 may be functional in this tumour but inactivated by high expression of mdm2

Of the five tumours with wild-type p53 sequence and negative or weakly positive p53 immunohistochemistry, two had HPV 16 expression (tumours 10, 19), one had high expression of mdm2 (tumour 11), one tumour had both (tumour 17) and one had neither. Of the two tumours with wild-type p53 and strongly positive p53 immunohistochemistry (tumours 9, 21), both had high expression of mdm2 indicating inactivation by mdm2. Thus, six of the seven tumours with wild-type p53 had inactivation of p53 either by HPV 16 infection or by overexpression of mdm2. Examples of mdm2 immunohistochemistry in tumours 17 and 21 are shown in Figure 3.

### DISCUSSION

In this paper we have shown that in recurrent SCCHN there is a high incidence of p53 mutation. Fifteen of the 22 tumours (68%) had either a p53 mutation or deletion. Of these, six were mis-sense mutations, whereas nine had non-sense inactivation. The immunohistochemistry results correlated very well with the mutations seen. Previous studies in primary head and neck cancer have shown an incidence of p53 mutation of 38–53% (Brachmann et al, 1992; Boyle et al, 1993; Mao et al, 1996; Olshan et al, 1997; Koh et al, 1998). It has been suggested that p53 mutation is an early event in head and neck carcinogenesis. Boyle et al (1993) showed an incidence of mutation of 19% in pre-invasive lesions increasing to 43% in invasive lesions. However, Chung et al (1993) showed that the frequency of p53 mutations did not increase with stage of disease with a reported incidence of 47% in stage I/II disease and 37% in stage III/IV disease. In our study, we have reported an incidence of p53 mutation in recurrent SCCHN of 68%, which is higher than that reported for primary disease. We did not carry out microdissection of tumour cells from surrounding normal cells in the preparation of tumour DNA so it is possible that some tumours with wild-type *p53* gene sequence are in fact false-negatives and therefore the incidence may be higher than 68%. Our figures (and those reported for primary disease by other studies) are also based upon sequencing exons 4–9 of the *p53* gene. Although 95% of mutations occur in these exons, some mutations occur in intron regions and also other exons. Therefore the incidence of p53 mutations in our study may be higher. Similarly, the incidence in previously published studies on primary head and neck cancer may also be slightly higher than 38–53%. It is also possible that previous studies in primary disease have underestimated the incidence of p53 mutation due to the heterozygous nature of primary tumours resulting in false-negative results on gene sequencing. In recurrent tumours, clonal expansion of tumour cells refractory to radiotherapy occurs and therefore the pickup rate for detecting p53 mutation may be more accurate. However, another possibility may be the induction of p53 mutations by radiotherapy treatment itself since radiation is a potent DNA damaging agent and induces single and multiple base pair deletions in DNA. It is difficult to distinguish between these two possibilities but one method could be to prepare molecular probes to the p53 mutation in each recurrent tumour and then to use these probes to screen the primary tumours to look for the mutation. This study is currently underway and will be reported separately.

p53 can be inactivated by HPV due to the ability of HPV E6 protein to bind to and promote the degradation of the product of the *p53* gene (Levine, 1990; zur Hausen, 1994). Indeed, inactivation of p53 by HPV E6 is the major mechanism of p53 inactivation in cervical carcinogenesis (zur Hausen, 1994) with approximately 80–90% of cervical cancers containing HPV DNA (Yoshikawa, 1991). Previous studies have shown HPV DNA in tumours of the

*© 2000 Cancer Research Campaign*

head and neck but its prevalence varies from 10–58% (Haraf et al, 1996; Paz et al, 1997; Koh et al, 1998; Miguel et al, 1998). A metanalysis by McKaig et al (1998) showed an overall incidence of 34.5% with the predominant type being HPV 16. The incidence also varies with tumour site with the highest prevalence being in tonsillar carcinoma (Paz et al, 1997). In our study in recurrent tumours of the head and neck, we found an overall incidence of 36% (8/22), all of which were HPV 16. These results were very similar to the meta-analysis figures by McKaig for primary tumours and this would suggest that HPV infection is not a major aetiological factor in recurrent disease. One of the limitations of our study, and of previously published studies, is that HPV detection is based upon PCR analysis of HPV DNA and not mRNA of HPV E6–E7 regions. It is possible that PCR analysis of HPV DNA can overestimate the presence of HPV due to cross-contamination resulting in false-positives. Since RNA is readily degradable, cross-contamination is less likely to result in false-positives. Another advantage of using RNA is that it proves that any HPV present in tumour cells is actually expressed at the mRNA level. Some studies have reported HPV mRNA detection using in situ hybridization of paraffin-embedded samples (Stoler et al, 1992). A more accurate method, however, is to use reverse transcription PCR (RT-PCR) as reported by Czegledy et al (1995). In our study, DNA was used because the tumour samples were too small to allow an adequate amount of RNA to be prepared. Of the eight tumours which were positive for HPV, five were in tumours with mutant p53 and three were in tumours with wild-type p53. These figures are similar to those in primary tumours as reported by Koh et al (1998) and suggests that p53 mutation and HPV positivity are not mutually exclusive mechanisms of p53 inactivation. This is in contrast to cervical carcinogenesis where p53 mutations are rare in cases with HPV but common in malignancies devoid of HPV infection (Park et al, 1990; Crook et al, 1991).

Another factor which can inactivate p53 is the cellular protein mdm2. Mdm2 inhibits the transcriptional activity of p53 by binding to the N terminus of p53 (Haupt et al, 1997) which in turn leads to the degradation of p53 via the ubiquitin degradation pathway. Thus amplification or overexpression of mdm2 can cause p53 inactivation (Oliner et al, 1992). In primary SCCHN, mdm2 overexpression is reported to be 40% (Matsumura et al, 1996). Girod et al (1995) showed an increase in mdm2 expression from premalignant to malignant lesions indicating that this may be a mechanism for inactivation of p53 early in head and neck carcinogenesis. Further evidence to suggest this was reported by Pruneri et al (1997), who showed that out of 17 laryngeal tumours overexpressing mdm2, ten of these also overexpressed p53 and all ten had wild-type p53 sequence. The importance of mdm2 overexpression in recurrent SCCHN is unknown. In the small group of tumours which we have studied, we have shown an incidence of 50% of which 5/11 were weakly positive and 6/11 were strongly positive for mdm2. Mdm2 overexpression was present in seven tumours with mutant p53 and in four tumours with wild-type p53, and this also suggests that p53 mutation and mdm2 overexpression are not mutually exclusive mechanisms of p53 inactivation. A particularly intriguing observation is that of the seven tumours which had wild-type p53 sequence, six were positive for either HPV infection, mdm2 overexpression or both. Thus in wild-type p53 tumours, the *p53* gene is inactivated by other mechanisms making the overall incidence of p53 inactivation in recurrent SCCHN in this study to be 95% (21 out of 22 tumours). It is unclear whether or not this overall incidence is greater than primary SCCHN since no study has ever examined p53 mutation, HPV infection and mdm2 overexpression in the same group of tumours. The observation that the incidence of HPV infection and mdm2 overexpression are similar to primary disease indicates that these factors have a limited role in the aetiology of recurrent SCCHN but may be more important in tumours in which the *p53* gene is not inactivated by mutation. We have shown that the incidence of p53 mutation in recurrent disease is higher than primary disease and have suggested that one possible reason for this could be due to DNA damage induced by radiation itself. We are currently carrying out a study to determine the importance of this observation using molecular probes to each individual p53 mutation to screen the primary tumour for the mutation.

The observation that recurrent disease has a very high incidence of p53 inactivation is clearly important as p53 is central to apoptosis induced by radiation and many chemotherapeutic agents. This may account for the poor response of these tumours to both reirradiation and chemotherapy. Therefore, new therapies which can restore p53 functionality, such as adenoviral mediated transfer of wild-type p53 (Roth et al, 1996), may be beneficial in this disease. Indeed, in vitro and in vivo studies using such a technique have demonstrated growth suppression in SCCHN cell lines (Liu et al, 1994, 1995; Clayman et al, 1995). Results of a phase I study in patients with recurrent SCCHN were also recently reported (Clayman et al, 1998) and this showed evidence of tumour necrosis in some patients. Other methods of restoring p53 function could be to use small molecules to inactivate the mdm2 protein (Bottger et al, 1998), or drugs which interfere with HPV E6 binding to wild-type p53 thus restoring function of p53. Recently, the selectively replicating adenovirus, Onyx-015, which targets cells with mutant p53, was reported (Bischoff et al, 1996; Heise et al, 1997). Results of a phase I study in patients with recurrent head and neck cancer using this virus appear to be promising (Ganly et al, 1997). Other approaches to the treatment of this disease may be to use drugs which act in a p53-independent manner. For example, Taxanes (Taxol) act by inducing p53-independent apoptosis and have been successful in the treatment of refractory cisplatin-resistant ovarian cancer, a disease where there is also a high incidence of p53 inactivation (Righetti et al, 1996; Buttitta et al, 1997). Catimel et al (1994) has shown this agent to be effective in recurrent SCCHN but there is no evidence yet that it has increased benefit over cisplatin–5-fluorouracil chemotherapy. However, newer agents which act in a p53-independent manner may prove to be more beneficial.

### REFERENCES

Bischoff JR, Kirn DH, Williams A, Heise C, Horn S, Muna M, et al (1996) A mutant adenovirus which selectively replicates in tumour cells with nonfunctional p53. *Science* **274:** 373–376

Bottger A, Bottger V, Sparks A, Liu WL, Howard SF and Lane DP (1997) Design of a synthetic mdm2-binding mini protein that activates the p53 response in vivo. *Curr Biol* **7:** 860–869

Boyle JO, Koch W, Hruban RH, van der Riet P and Sidransky D (1993) The incidence of p53 mutations increases with progression of head and neck cancer. *Cancer Res* **53**: 4477–4480

Brachman DG, Graves D, Vokes E, Beckett M, Haraf D, Montag A, et al. (1992) Occurrence of p53 gene deletions and human papilloma virus infection in human head and neck cancer. *Cancer Res* **52**: 4832–4836

Buttitta F, Marchetti A, Gadducci A, Pellegrini S, Morganti M, Carnicelli V, et al. (1997) p53 alterations are predictive of chemoresistance and aggressiveness in ovarian carcinomas: a molecular and immunohistochemical study. *Br J Cancer* **75**: 230–235

© 2000 Cancer Research Campaign

Catimel G, Verweij J, Mattijssen V, Hanauske A, Piccart M, Wanders J, et al (1994) Docetaxel (taxotere): an active drug for the treatment of patients with advanced squamous cell carcinoma of the head and neck. *Ann Oncol* **5**: 533–537

Czegledy J, Iosif C, Hanson BG, Evander M, Gergely L and Wadell G (1995) Can a test for E6/E7 transcripts of human papillomavirus type 16 serve as a diagnostic tool for the detection of micrometastases in cervical cancer. *Int J Cancer (Pred Oncol)* **64**: 211–215

Chung KY, Mukhopadhyay T, Kim J, Casson A, Ro JY, Goepfert H, et al (1993) Discordant p53 gene mutations in primary head and neck cancers and corresponding second primary cancers of the upper aerodigestive tract. *Cancer Res* **53**: 1676–1683

Clavel M, Vermorken JB, Cognetti F, Cappelaere P, de Mulder PHM, Schornagel JH, et al (1994) Randomised comparison of cisplatin, methotrexate, bleomycin and vincristine (CABO) versus cisplatin and 5-fluorouracil versus cisplatin in recurrent or metastatic squamous cell carcinoma of head and neck. *Ann Oncol* **5**: 521–526

Clayman GL, El-Naggar AK, Roth JA, Zhang WW, Goepfert H, Taylor DL, et al (1995) In vivo molecular therapy with p53 adenovirus for microscopic residual head and neck squamous carcinoma. *Cancer Res* **55**: 1–6

Clayman GL, El-Naggar AK, Merritt J, Bruso P, Roth JA, Lippman S, et al (1998) Adenovirus mediated p53 gene transfer in patients with advanced recurrent head and neck squamous cell carcinoma. *J Clin Oncol* **16**: 2221–2232

Crook T, Wrede D and Vousden KH (1991) p53 point mutations in HPV negative human cervical carcinoma cell lines. *Oncogene* **6**: 873–875

Cutilli T, Papola F, Di Emidio P, Leocata P and Corbacelli A (1998) p53 mutations and chemoresistance in oromaxillofacial squamous cell carcinomas. The results of a molecular genetics study of p53 in metastatic oromaxillofacial tumours and an evaluation of the response to neoadjuvant chemotherapeutic treatment. *Minerva Stomatol* **47**: 1–9

Forastiere AA (1994) Overview of platinum chemotherapy in head and neck cancer. *Semin Oncol* **21**: 20–27

Ganly I, Kirn D, Rodriguez GI, Soutar D, Eckhardt G, Otto R, et al (1997) Phase I trial of intratumoural injection with an E1B attenuated adenovirus, ONYX-015, in patients with recurrent p53(–) head and neck cancer. *J Clin Oncol* **16**: 1362

Girod SC, Cesarz D, Fischer U and Krueger GR (1995) Detection of p53 and mdm2 protein expression in head and neck carcinogenesis. *Anticancer Res* **15**: 1453–1457

Haraf DJ, Nodzenski E, Brachman D, Mick R, Montag A, Graves D, et al (1996) Human papilloma virus and p53 in head and neck cancer: clinical correlates and survival. *Clin Cancer Res* **2**: 755–762

Haupt Y, Maya R, Kazaz A and Oren M (1997) Mdm2 promotes the rapid degradation of p53. *Nature* **387**: 296–299

Heise H, Sampson-Johannes A, Williams A, McCormack F, Von Hoff DD and Kirn DH (1997) Onyx-015, an E1B gene-attenuated adenovirus, causes tumour specific cytolysis and antitumoural efficacy that can be augmented by standard chemotherapeutic agents. *Nat Med* **3**: 639–645

Huang LC, Clarkin KC and Wahl GM (1996) Sensitivity and selectivity of the DNA damage sensor responsible for activating p53-dependent G1 arrest. *Proc Natl Acad Sci USA* **94**: 4827–4832

Jones KR, Lodge-Rigal RD, Reddick RL, Tudor GE and Shockley WW (1992) Prognostic factors in the recurrence of stage I and II squamous cell cancer of the oral cavity. *Arch Otolaryngol Head Neck Surg* **118**: 483–485

Kao CC, Yew PR and Berk AJ (1990) Domains required for in vitro association between the cellular p53 and the adenovirus 2 E1B 55K proteins. *Virology* **179**: 806–814

Koh JY, Cho NP, Kong G, Lee JD and Yoon K (1998) p53 mutations and human papilloma DNA in oral squamous cell cancer: correlation with apoptosis. *Br J of Cancer* **78**: 354–359

Lechner MS (1992) Human papillomavirus E6 proteins bind p53 in vivo and abrogate p53-mediated repression of transcription. *Embo J* **11**: 3045–3052

Levine AJ (1990) The p53 protein and its interactions with the oncogene products of the small DNA tumour viruses. *Virology* **177**: 419–426

Liu TJ, Zhang WW, Taylor DL, Roth JA, Goepfert H and Clayman GL (1994) Growth suppression of human head and neck cancer cells by the introduction of a wild type p53 gene via a recombinant adenovirus. *Cancer Res* **54**: 3662–3667

Liu TU, El-Nagger AK, McDonnell TJ, Steck KD, Wang M, Taylor DL, et al (1995) Apoptosis induction mediated by wild-type p53 adenoviral gene transfer in squamous cell carcinoma of the head and neck. *Cancer Res* **55**: 117–122

Lowe SW, Riley HE, Jack T and Honsman DE (1993) p53 dependent apoptosis modulates the cytotoxicity of anticancer agents. *Cancer Res* **54**: 3500–3505

Mao EJ, Schwartz SM, Daling JR, Oda D, Tickman L and Beckmann AM (1996) Human papilloma viruses and p53 mutations in normal pre-malignant and malignant oral epithelia. *Int J Cancer* **69**: 152–158

Matsumura T, Yoshihama Y, Kimura T, Shintani S and Alcalde RE (1996) p53 and mdm2 expression in oral squamous cell carcinoma. *Oncology* **53**: 308–312

McIlwrath AJ, Vasey PA, Ross GM and Brown R (1994) Cell cycle arrests and radiosensitivity of human tumour cell lines: dependence on wild type p53 for radiosensitivity. *Cancer Res* **54**: 3718–3722

McKaig RG, Baric RS and Olshan AF (1998) Human papillomavirus and head and neck cancer: epidemiology and molecular biology. *Head Neck* **20**: 250–265

Miguel RE, Villa LL, Cordeiro AC, Prado JC, Sobrinho JS and Kowalski LP (1998) Low prevalence of human papillomavirus in a geographic region with a high incidence of head and neck cancer. *Am J Surg* **176**: 428–429

Nabel GJ, Nabel EG, Yang ZY, Fox BA, Plautz GE, Gao X, et al (1993) Direct gene transfer with DNA-liposome complexes in melanoma: expression, biological activity, and lack of toxicity in humans. *Proc Natl Acad Sci USA* **90**: 11307–11311

Nickers P, Kunkler I and Scalliet P (1997) Modern brachytherapy: current state and future prospects. *Eur J Cancer* **33**: 1747–1751

Oliner JD, Kinzler KW, Meltzer PS, George D and Vogelstein B (1992) Amplification of a gene encoding a p53 associated protein in human sarcomas. *Nature* **358**: 80–83

Olshan AF, Weissler MC, Pei H and Conway K (1997) p53 mutations in head and neck cancer: new data and evaluation of mutational spectra. *Cancer Epidemiol Biomarkers Prev* **6**: 499–504

Park T, Wilezynski SP, Paquette RL, Miller CW and Koeffler HP (1994) p53 mutations in HPV negative cervical carcinoma. *Oncogene* **9**: 205–210

Paz IB, Cook N, Odom-Maryon T, Xie Y and Wilczynski SP (1997) Human papillomavirus (HPV) in head and neck cancer. An association of HPV16 with squamous cell carcinoma of Waldeyer's ring. *Cancer* **79**: 595–604

Pruneri G, Pignataro L, Carboni N, Luminari S, Capaccio P, Neri A, et al (1997) Mdm2 oncoprotein overexpression in laryngeal squamous cell carcinoma: association with wild-type p53 accumulation. *Mod Pathol* **10**: 785–792

Righetti SC, Torre GD, Pilotti S, Menard S, Ottone F, Colnaghi MA et al (1996) A comparitive study of p53 gene mutations, protein accumulation and response to cisplatin-based chemotherapy in advanced ovarian carcinoma. *Cancer Res* **56**: 689–693

Roth JA (1996) Retrovirus mediated wild type p53 gene transfer to tumours of patients with lung cancer. *Nature Med* **2**: 985–991

Scheffner M, Werness BA, Huibregtse JM, Levine AJ and Howley PM (1990) The E6 oncoprotein encoded by human papillomavirus types 16 and 18 promotes the degradation of p53. *Cell* **63**: 1129–1136

Stoler MM, Rhodes CR, Whitbeck A, Wolinsky SM, Chow LT and Broker TR (1992) Human papillomavirus type 16 and 18 gene expression in cervical neoplasms. *Hum Pathol* **23**: 117–128

Snow GB (1989) Evaluation and staging of the patient with head and neck cancer. Churchill Livingstone, New York*:* 17–38

Yeudall WA and Campo MS (1991) Human papillomavirus DNA in biopsies of oral tissues. *J Gen Virol* **72**: 173–176

Yoshikawa H, Kawana T, Kitagawa K, Mizuno M, Yoshikura H and Iwamoto A (1991) Detection and typing of multiple genital human papillomaviruses by DNA amplification with consensus primers. *Jpn J Cancer Res* **82**: 524–531

zur Hausen H (1994) Molecular pathogenesis of cancer of the cervix and its causation by specific human papillomavirus types. *Curr Topics Microbiol Immunol* **186**: 131–156

*© 2000 Cancer Research Campaign*
